# Orthostatic Hypotension in Patients with Parkinson's Disease and Atypical Parkinsonism

**DOI:** 10.1155/2014/475854

**Published:** 2014-02-02

**Authors:** Seyed-Mohammad Fereshtehnejad, Johan Lökk

**Affiliations:** ^1^Division of Clinical Geriatrics, Department of Neurobiology, Care Sciences and Society (NVS), Karolinska Institutet, Novum 5th Floor, 141 86 Stockholm, Sweden; ^2^Firoozgar Clinical Research Development Center (FCRDC), Firoozgar Hospital, Iran University of Medical Sciences, Tehran 15937-48711, Iran; ^3^Department of Geriatric Medicine, Karolinska University Hospital, 141 86 Stockholm, Sweden

## Abstract

Orthostatic hypotension (OH) is one of the commonly occurring nonmotor symptoms in patients with idiopathic Parkinson's disease (IPD) and atypical parkinsonism (AP). We aimed to review current evidences on epidemiology, diagnosis, treatment, and prognosis of OH in patients with IPD and AP. Major electronic medical databases were assessed including PubMed/MEDLINE and Embase up to February 2013. English-written original or review articles with keywords such as “*Parkinson's disease*,” “*atypical parkinsonism*,” and “*orthostatic hypotension*” were searched for relevant evidences. We addressed different issues such as OH definition, epidemiologic characteristics, pathophysiology, testing and diagnosis, risk factors for symptomatic OH, OH as an early sign of IPD, prognosis, and treatment options of OH in parkinsonian syndromes. Symptomatic OH is present in up to 30% of IPD, 80% of multiple system atrophy (MSA), and 27% of other AP patients. OH may herald the onset of PD before cardinal motor symptoms and our review emphasises the importance of its timely diagnosis (even as one preclinical marker) and multifactorial treatment, starting with patient education and lifestyle approach. Advancing age, male sex, disease severity, and duration and subtype of motor symptoms are predisposing factors. OH increases the risk of falls, which affects the quality of life in PD patients.

## 1. Introduction

Orthostatic hypotension (OH) is the most common symptom of cardiovascular autonomic dysfunction in parkinsonian patients [1, 2], which can affect both patients with idiopathic Parkinson's disease (IPD) and atypical parkinsonism (AP). In general, OH refers to a fall in systolic blood pressure of at least 20 mmHg and diastolic blood pressure of at least 10 mmHg on standing or head-up tilt [3]; its prevalence in PD varies between 9.6% and 58% [4, 5].

OH prevalence correlates with disease duration and could also be a result of PD medications, which lead to gait instability, generalised weakness, fatigue, a higher risk of falls, and reduced cognitive performance. It may herald the onset of PD years before motor features become apparent. Together with other nonmotor symptoms, OH may gradually and critically impact quality of life in parkinsonian patients [6, 7]. Although some original studies have been previously performed on this topic, there are still arguments and gap of knowledge on many aspects of OH from the prevalence to treatment strategies. Nowadays, OH causes a major management problem in parkinsonian patients, which necessitates further evaluation and improvement in our knowledge.

In attempting to address some of these knowledge gaps, this review aimed to summarise current evidences on different aspects of OH in parkinsonian patients including epidemiology, aetiology, diagnosis, predisposing factors, prognosis, and treatment.

## 2. Methods

We searched relevant databases to find appropriate articles on OH in IPD and/or AP. For this purpose, major electronic medical databases were assessed including PUBMED/MEDLINE and EMBASE up to February 2013. Combined search terms such as “*Parkinson's disease*,” “*atypical parkinsonism*,” and “*orthostatic hypotension*” were entered to look for English-written original or review papers. Afterwards, articles were retrieved, and references were also searched for relevant manuscripts containing the abovementioned keywords. We consider only published or in-press peer-reviewed articles to provide qualified evidences for this narrative review paper. After reviewing of the relevant papers with different types such as review articles, original researches, and case reports, current knowledge on this topic was categorised and presented in several sections consisting of “definition,” “symptoms,” “epidemiology,” “pathophysiology,” “diagnosis,” “prodromal OH,” “predisposing factors,” “prognosis,” and “treatment.”

## 3. Results

Based on the literature review about the main topic of interest, orthostatic hypotension in parkinsonian patients, the following aspects were identified to have both research importance and clinical implications.

### 3.1. Definition

Based on the agreement of the Consensus Committee of the American Autonomic Society and the American Academy of Neurology, OH has been defined as a sustained fall of ≥20 mmHg in systolic or ≥10 mmHg in diastolic blood pressure within 3 min of active standing or head-up tilt to at least 60°. In the recent revision of the consensus statement, a systolic fall of 30 mmHg was defined as OH for patients with an abnormally high supine blood pressure [3]. However, some symptomatic patients may have a much greater fall in blood pressure on standing [1].

It must be considered that some PD patients might have the underlying pathology of OH but no apparent symptoms. Findings from a transcranial Doppler (TCD) study show that the impairment of cerebral vasomotor reactivity (VMR) is even more common than detectable OH in PD patients [8]. The prevalence rate of asymptomatic OH in PD has been estimated as 20–50% in another published report. This study also showed that OH might occur even after the currently recommended duration of 3 min for tilt testing [9]. Some researchers now impugn the accuracy of the current criteria for OH definition in which blood pressure is examined after 3 min of gravitational stimulus [9]. Current criteria may miss OH in some parkinsonian patients as symptoms of OH secondary to autonomic failure characteristically appear after tilting or standing, which are relieved upon sitting or lying flat [10].

### 3.2. Symptoms

It is not still quite clear why OH is asymptomatic in a subgroup of PD patients. However, OH may present some nonspecific general symptoms in parkinsonian patients consisting of giddiness, dizziness, empty-headedness, visual disturbances, unconsciousness, weakness, falls, syncope, and even nausea or pain, mostly while standing [11, 12]. It has already been demonstrated that some OH symptoms such as dizziness, visual disturbances, impaired cognition, and fainting occur when the decline in blood pressure induces impairment in cerebral perfusion [13], and causes a failure in cerebrovascular autoregulation [14].

In one recently published paper, Ha et al. reported that older age, more advanced disease, longer duration of PD, and a wider range of sitting blood pressure increased the risk of symptomatic OH [15]. Predictably, symptomatic OH is more common in PD patients with posture and gait instability phenotype compared to those with the tremor-dominant variety of the disease [16, 17].

### 3.3. Diagnosis

Nowadays, several methods are used to diagnose and/or assess OH in parkinsonian patients such as physical examination, neuroimaging techniques, and subjective or objective scales and questionnaires (Table 2).

During physical examination, OH is diagnosed using a gravitational stimulus such as tilting or standing. Physicians used to assess OH by recording the changes in blood pressure using a sphygmomanometer after the PD patient had been standing for 3 min from the supine position. However, in a research setting, OH is more often checked by means of a tilt table [9]. Demonstrating the fact that OH is more likely to occur after tilting than standing [15], this type of clinical examination may result in underestimation of OH in PD patients.

In head-up tilt-table testing (HUT), the subjects are tilted to a 60-degree upright position within 15 seconds using a head-up tilt table. As a simple, noninvasive, and informative method, the tilt-table test is performed by having the patient lie flat on a special bed or table with special safety belts and a footrest while monitoring for blood pressure is done. The bed or table is then elevated to an almost standing position (60-to-80-degree vertical angle) to simulate the patient standing up from a lying position. The blood pressure is measured during the test to evaluate its probable drop during this positional change. Patients are defined as having OH if the standing systolic blood pressure falls by at least 20 mmHg or ≥10 mmHg occurred in diastolic blood pressure [3]. The Valsalva manoeuvre is another physical examination that can be used to assess autonomic function in haemodynamics in PD patients. It has been shown that findings from this manoeuvre are highly sensitive and reproducible for the assessment of arterial baroreflex [18], the function which is attenuated in PD. In the Valsalva manoeuvre, patients are asked to exhale into a mouthpiece at an expiratory pressure of 40 mmHg for 15 seconds. Blood pressure and RR intervals (from the corresponding ECG) are measured during the manoeuvre by tonometry, using a noninvasive blood pressure monitoring system. The duration of the Valsalva manoeuvre is divided into four phases and several indexes, including the baroreceptor reflex sensitivity (BRS), which are measured within specific phases. Analyses of data on blood pressure, RR interval, and BRS allow conclusions to be drawn about the autonomic haemodynamics of the patients [19–21]. Spectral analysis of the RR interval and systolic blood pressure are commonly used to measure cardiac autonomic activity, representing cardiac sympathovagal interaction, and their findings are variably impaired in PD [22]. Even more, these findings of heart rate variability may be an early manifestation of PD, which might be useful in the assessment of the rate of disease progression and the efficacy of medication [23]. Using spectral analysis of RR interval and systolic blood pressure, one study showed reduced absolute values of both the high- and low-frequency spectral components in PD patients compared with age-matched healthy subjects [24]. Another study demonstrated a higher systolic blood pressure, lower low- to high-frequency ratio and low-frequency systolic blood pressure in PD patients with symptomatic OH compared to control subjects using spectral analysis [25]. Interestingly, they also reported that the increase in heart rate, low- to high-frequency ratio, and low-frequency systolic blood pressure was blunted in PD patients with or without symptomatic OH compared to the control group during tilt [25]. These findings further confirm that spectral analysis of heart rate may be useful in early diagnosis of orthostatic intolerance regardless of a recognized orthostatic hypotension in the history of PD patients [25].

As shown in the Movement Disorders Society Task Force report, OH is usually contained within the list of questions that record either the whole nonmotor or dysautonomia symptoms. From another point of view, while some scales provide information on the severity and/or frequency of OH-related symptoms, others only ask for the presence or absence of OH symptoms [26]. Table 2 lists the names of well-validated scales that include items to record the severity and/or frequency of OH-related symptoms. Other than the level of symptoms, it must be noted that not all OH-related symptoms are recorded in all of these scales and the medical terminology applied to express them might also differ. For instance, the Orthostatic Grading Scale [27] and L-threo-DOPS [28, 29] measure “*maximal standing time*,” whereas other scales such as the AUTonomic SCale for Outcomes in PArkinson's Disease (SCOPA-AUT) [30] and the Composite Autonomic Symptom Scale (COMPASS) [31] directly asked for “*faintness*” or “*syncope*.” Faintness, dizziness, and light-headedness are the most frequent orthostatic symptoms that are recorded in most of these rating scales; however, “*decreased hearing*” is only rated in the scale used by Senard et al. [32] and “*difficulty thinking*” is only measured by the SCOPA-AUT [30] and the COMPASS [31]. More detailed comparisons are presented in the Movement Disorders Society Task Force report [26]. Conclusively, they recommended SCOPA-AUT [30] and COMPASS [31] as the clinimetrically proved scales; however, other questionnaires are also suggested as valid screening tools for OH in PD. As a well-known commonly used questionnaire, the Unified Parkinson's Disease Rating Scale (UPDRS) has only one item addressing OH. This item is in the part IV of the UPDRS scale on “complications of therapy” and was reported as a low sensitivity screening tool for OH [26]. In addition to the scales listed in Table 2, the Unified Multiple System Atrophy Rating Scale (UMSARS) is also applied to assess different symptoms in MSA, including autonomic problems as a semiquantitative tool [33], but this has never been used in IPD patients [26].

According to underlying pathophysiology of OH in PD, nuclear neuroimaging techniques are applicable in suspected patients. Almost all patients with PD and OH have markedly reduced sympathetic noradrenergic innervation of the left ventricular myocardium, which could be evaluated with single-photon emission computed tomography and ^123^I-meta-iodobenzylguanidine (MIBG) uptake. Interestingly, this finding is not seen in MSA patients with OH and could be helpful for differential diagnosis [34, 35]. In order to perform cardiac ^123^I-MIBG scintigraphy, firstly the tracer ^123^I-MIBG is injected intravenously in an amount of 111 MBq for the patients. Then, region-of-interest (ROI) analysis is applied to evaluate the ^123^I-MIBG uptake, and the ratio of the average pixel count in the heart to that in the mediastinum is calculated and reported after the early (15 min) and delayed (3 hour) phases [36]. This parameter is used to judge the cardiac sympathetic noradrenergic innervation. Another paraclinical assessment tool that has potential application in the study of OH in parkinsonian patients is transcranial Doppler (TCD) ultrasonography. As a noninvasive technique, TCD measures blood flow velocities of the main intracranial vessels, such as the middle cerebral artery, and is a broadly applied tool for evaluating the cerebrovascular reactivity in neurological disorders including PD. To evaluate dysautonomia, vasomotor reactivity, and cerebral haemodynamics in parkinsonian patients, TCD is usually used in combination with one of the standardised methods applied to activate the sympathetic adrenergic innervation system. These methods include the tilt-table test [37], head-up tilt test [38], thigh cuff release test [39], carbon dioxide test [8], Diamox injection [40], or the cold pressure test (CPT) [41, 42]. These techniques make it possible to identify even asymptomatic OH patients.

### 3.4. Epidemiology

So far, many epidemiologic studies have been performed to estimate the prevalence rate of OH in PD or other parkinsonian syndromes. One important aspect of these epidemiologic surveys refers to study design. While most of these studies have been designed as cross-sectional projects to assess the point prevalence of OH, there are a few longitudinal cohorts, which were able to report the incidence rate of OH during disease followup. The point prevalence shows a snapshot of the proportion of PD patients with OH that exists in a defined PD population at one specific time point. Therefore, it does not contain any further information on the relationship between OH incidence and disease progression.

In one longitudinal cohort study, almost 48% of PD patients represented symptomatic OH during a 20-year follow-up period [43]. Expectedly, the point prevalence estimations of cross-sectional reports are often lower. In general, published estimates of OH in idiopathic PD range between 9.6% and 47% [4, 32, 44, 45] and are even as high as 58% [5]. Recently, in a meta-analysis of 25 recruited studies, a pooled point prevalence of 30.1% (95% CI: 22.9% to 38.4%) was estimated for OH in patients with PD [46].

The discrepancies in prevalence rate estimation of OH in PD stem from several factors such as selection criteria, study population, study design (cross-sectional versus longitudinal), the criteria used to define OH, and the method of BP measurement [47]. The most important factor refers to the diagnostic methods and/or criteria that are applied to define OH in a PD population. For instance, not all studies have used the strict standard definition as a fall of 20 mmHg systolic or 10 mmHg diastolic blood pressure within 3 min of tilting or standing [9]. Even if most of the studies used this definition, the examination setting, such as tilting angle, might also differ, which could be considered another source of diversity. While some researchers used 45 degrees to ensure that the participants could tolerate the full course of head-up tilting [48], an angle of 60 to 70 degrees of tilting is usually required to provoke OH with a higher diagnostic value [49, 50]. As a consequence, not only do the prevalence data vary between studies, but also associated risk factors and probable effects of OH during the PD course are also dependent on the exact definitions adhered in that specific survey.

Rather than idiopathic PD, available data regarding the frequency of OH in atypical parkinsonian syndromes are not sufficient and most of the reports lack appropriate sample size.  Table 1 summarises the frequency rates of OH in different types of parkinsonian syndromes estimated from some of the important recent surveys on this issue. Although the number of recruited patients with atypical PD is considerably lower than IPD and a definitive conclusion is limited, it seems that OH is most frequent in MSA. The prevalence of OH varies from as low as 0–7% in CBD, to the higher rates in PSP, VP, DLB, and IPD and to as high as 81% in MSA (Table 1). This noticeable high-frequency rate of symptomatic OH in MSA could present even in the early stage of the disease and is expected due to the well-known autonomic failure in MSA [15].

### 3.5. Pathophysiology

In general, a degree of damage to the postganglionic sympathetic efferences is suggested as the main cause of dysautonomia in PD, while these postganglionic lesions are not found in patients with MSA [57]. Based on current evidences, three main components work together to induce OH in PD, including noradrenergic denervation in both cardiac and extracardiac regions and arterial baroreflex failure as the third determinant [58]. This underlying pathophysiology is believed to act independently of the nigrostriatal dopamine depletion [58, 59]. Although direct evidences come from dementia with Lewy bodies, the myocardial sympathetic denervation was shown to associate with decreased vesicular uptake of catecholamines [60], in a process that might start years before motor involvement in PD. This pathology is highly typical as all of the PD patients with OH noticeably suffer from reduced sympathetic noradrenergic innervation of the left ventricular myocardium [61], which demonstrates a strong link between sympathetic cardiac denervation and OH in PD. Even Jain and Goldstein recommend that one can exclude PD diagnosis in central neurodegenerative disorder with OH when normal noradrenergic innervation is reported for myocardium in imaging evaluations [58]. However, one study using positron emission tomography (PET) scanning showed that this denervation is profound in the heart but also could be detected in extracardiac organs such as the renal cortex and thyroid gland [62].

The third part of the pathophysiological puzzle of OH in PD, arterial baroreflex failure, involves both the cardiovagal and sympathoneural limbs, which results in an obviously lower extent of decrease in the time between heartbeats (interbeat interval) during the Valsalva manoeuvre [58]. This pathophysiologic base could also induce postprandial hypotension, instability of blood pressure, supine hypertension, and possibly fatigue and exercise intolerance [58]. As a consequence, coexistent supine hypertension and orthostatic hypotension may occur in some PD patients resulting from the malfunctioning of baroreflex system. Therefore, clinicians should be aware of this possibility in PD patients presenting symptoms of orthostatic intolerance, especially in those with a large supine standing fluctuation in blood pressure [63].

From a broader point of view, the baseline process of synuclein accumulation in intraneural Lewy bodies of the limbic cortex, frontal neocortical areas [64, 65], and the peripheral autonomic nervous system [66] could provide evidence to explain the more rapid cognitive decline observed in PD patients suffering from OH [66, 67].

Despite the fact that the underlying cause for OH is independent of dopaminergic deficit, PD-related medications such as selegiline and dopamine agonists also contribute to the OH [68–70]. OH is further exacerbated by the use of dopaminergic drugs, such as levodopa, which has vasodilative effects via the renal and splanchnic vasculature [71, 72]. Consequently, selegiline may also augment the central hypotensive effect of levodopa by increasing dopamine availability through MAO-B inhibition [73], and it was shown that the withdrawal of selegiline suppresses the orthostatic blood pressure reaction in advanced PD patients [68].

### 3.6. Prodromal OH

OH is usually considered a symptom that typically develops and progresses during the later course of PD. Nowadays, it is believed that dysautonomia may herald the onset of PD even before motor symptoms manifest [15]. Also, OH can even precede PD diagnosis in some patients, which outlines OH as a potential prodromal marker in parkinsonian syndromes [74]. It has been shown that up to 60% of PD patients with OH had already suffered from the symptom in the early phase of PD [75]. Although prodromal OH in parkinsonian syndromes is mostly believed to be in favour of MSA due to its well-known autonomic failure [76–78], rising evidences suggest that this feature could precede PD as well. Even in some postmortem studies, early OH was recorded in autopsy-proven PD patients misdiagnosed as MSA [79, 80]. On the other hand, another study showed that one-third of pathologically proven MSA patients were mislabelled as PD during their life [81]. Dealing with the exact timing for the beginning of OH is always the subject of debate, and there is also a lack of pathologic postmortem evidence to differentiate between IPD and MSA. However, a neuroimaging technique that traces 6-[^18^F]fluorodopamine-derived radioactivity in the cardiac sympathetic system could assist in this issue. Based on evidences from postmortem studies, a marked decrease in its radioactivity throughout the left ventricular myocardium showing loss of postganglionic sympathetic noradrenergic nerves may exclude MSA [75].

Pathologically, this is now a question of interest whether loss of cardiac sympathetic nerves could precede the main nigrostriatal dopamine depletion in PD or not. If so, then symptoms such as OH could clinically present or at least be discovered years before the motor features of the disease start. Based on the Braak schema [82] of the pathologic development of PD, cardiac sympathetic denervation could be even found in PD patients without OH during the early phase of disease [58]. Although there is a gap of large sample size evidences, the idea has been proven in some case studies. By neuroimaging assessment, cardiac sympathetic denervation has been shown in one patient four years before PD onset [83] and in another with symptomatic OH five years before motor features [84]. More interestingly, such damage in cardiac sympathetic nervous system was reported in patients with OH who later developed a parkinsonism syndrome [75].

### 3.7. Predisposing Factors

To date, several factors have been found to relate to the presence of OH in PD, such as advancing age, male gender, disease severity or duration [36, 85–87], and clinical subtype of motor symptoms [22, 88]. PD duration has been shown to be a stronger determinant for OH compared to the severity of disease [89]. In addition, there are number of general predisposing factors for OH, which are not specific for PD. These factors include dehydration, deconditioning, poor nutrition, and aging-related changes [1]. Heat, food ingestion, alcohol, exercise, and activities that increase intrathoracic pressure (e.g., defecation and coughing) [57] must be also taken into consideration while evaluating a PD patient with OH.

Some of the antiparkinsonian medications could either cause or worsen OH. Levodopa could induce OH by reducing the stroke volume, cardiac output, and systemic vascular resistance [9, 90]. As previously mentioned, selegiline and dopamine agonists also could play a role in causing OH in PD patients [68–70]. However, evidences regarding the role of PD medication and OH are controversial. In one study on 55 PD patients, OH was not related to either dopaminergic medication such as levodopa or ergot and nonergot dopamine agonists [36]. Apart from PD-specific medications, some other drugs could predispose OH in PD patients, such as the chronic use of tricyclic antidepressants; antihypertensive agents and diuretics; vasodilators like nitroglycerin, hydralazine, calcium channel blockers, and tizanidine (Zanaflex) [1]. A recent study on 103 PD patients showed that polypharmacy, intake of diuretics or amantadine increase the risk of OH after statistical adjustment for confounding factors, whereas entacapone was found to be a protective factor [91]. Whether as a proxy for several comorbidities in frail elderly PD patients or due to pharmaceutical interaction, polypharmacy seems to be an important determinant to increase the risk of OH. Even a direct correlation has been shown between the number of medications and episodes of OH in elderly as well as PD patients [91, 92].

### 3.8. Prognosis

As can be seen from general evidences, OH is associated with increased mortality and morbidities in middle-aged adults and the elderly [93, 94] including stroke and cognitive decline [95].

Specifically in PD, OH is associated with postural sway [47] and an increased risk of falls [96]. There are also evidences suggesting that impaired attention [2] and visual episodic memory [97] are more frequent in PD patients suffering from OH. In one study on 44 PD patients, the presence of OH was shown to be related to greater levels of motor disability (specifically gross motor function), more difficulty with tasks that require postural changes or prolonged mobility, and a decline in cognitive function with lower MMSE score [98]. However, the effect of OH on cognition in PD patients is still conflicting, as some reports show no marked impairment of cognitive function when OH is present [44, 99]. Overall, it seems important to consider which subset of cognition is of interest; as can be seen from the aforementioned evidences, visual episodic memory and social cognition are more likely to be impaired in PD patients with OH.

It has already been demonstrated that OH considerably impairs the quality of life in PD patients [100], via an effect that could be implied either directly or indirectly through cognitive impairment, frequent falls, or other accompanied symptoms. The effect of OH on quality of life in parkinsonian patients is a two-dimensional causal relationship, as it has been shown that early diagnosis and symptomatic treatment of OH can greatly improve the quality of life in PD patients and, even in the case of pathologically elevated blood pressure, probably decrease cardiovascular mortality [101].

### 3.9. Treatment

Treatment of OH should be multifactorial, always starting with patient education and lifestyle approaches [15]. Hence, physical activity is a major cornerstone in OH treatment and patients must be urged to move and exercise to their full capacity. When moving from sitting or lying down, slowness in movements is prompted to avoid a fall in blood pressure [102]. Patients should change their eating habits from three meals a day to more frequent, smaller, and less carbohydrate-based meals, as well as having adequate hydration with at least 2 litres/day [103]. An increased intake of salt aiming at more than 8 grams daily can be tried, thereby increasing the plasma volume and blood pressure. By using compressive long stockings, a change in blood pooling from the lower to the upper parts of the body might elevate blood pressure and have some positive subjective effects on orthostatic coexisting unsteadiness and dizziness [104]. Raising the head of the bed on blocks, thereby improving the endocrinological regulatory system, should be tried before initiating pharmacological treatment [105]. When it comes to pharmacological treatment, blood pressure lowering drugs should first be reassessed and/or stopped. Naturally, the total dopaminergic load must be also reevaluated and, if possible, reduced, as this affects blood pressure. This being done with insufficient effects, sympathomimetic drugs like dihydroergotamine and/or etilefrine can be introduced in increasing/high doses [106]. If these provide unsatisfactory effects, the mineralocorticoid fludrocortisone, 1-2 mg daily, can be tried with some caution due to a risk of causing oedema following its plasma volume expansion [78]. The alpha-1-agonist midodrine in doses of 5–40 mg daily can be another quite powerful option, as can the use of the noradrenaline-like prodrug droxidopa [107]. There are also some reports of the antidiuretic hormone desmopressin and the peripheral dopamine blocker domperidone having effects on OH in PD patients [108].

However, the coexistence of OH and supine hypertension presents some additional problems when treating OH in affected PD patients. Using blockers of alpha-2 adrenoceptors such as yohimbine, which increases the release of norepinephrine and dopamine, can worsen supine hypertension. The same issue also exists when an indirectly acting sympathomimetic amine such as tyramine is given with a monoamine oxidase inhibitor [58].

## 4. Conclusion

This review summarises some of the important evidences, discrepancies, and issues on different aspects of OH in parkinsonian patients, such as epidemiology, pathophysiology, diagnosis, predisposing factors, prognosis, and treatment. Studies show that OH is quite common in PD and its timely diagnosis may need further assessments (such as paraclinical evaluations and/or blood pressure monitoring) beyond the recommended 3 min postural challenge that is currently used in clinical practice, as this could potentially miss some asymptomatic cases. Physicians must consider evaluating blood pressure in PD patients not only in the conventional sitting posture but also in different positional settings to be able to detect OH.

Our present knowledge of OH in parkinsonian syndromes highlights its important role in better understanding of PD aetiology, as well as its marked effect on quality of life of the parkinsonian patients. Educating patients and their families about OH symptoms could help in the early detection and better management of OH, which may improve their quality of life. There are still huge gaps in appropriate evidences and knowledge of several aspects of OH in parkinsonian patients, which emphasises the need for further studies on this topic. Inevitably, any progress in our knowledge about predisposing factors, diagnostic tools, and treatment options for OH might assist in the early detection of this potentially treatable cause to prevent falls in elderly parkinsonian patients. However, even based on current evidences that show the negative impacts of this common problem on different aspects of PD-specific quality of life (i.e., gross motor, balance, and cognitive function), it seems logical to routinely screen PD patients for OH.

## Figures and Tables

**Table 1:** Prevalence of symptomatic orthostatic hypotension in different types of parkinsonian syndromes.

Type of Parkinsonian syndromes	Reference	Sample size	Type of evidence	Prevalence rate (%)
Idiopathic Parkinson's disease (IPD)	[46]	5070	Meta-analysis	30.1
	[15]	1125	Cross-sectional	18
	[45]	3414	Multicentre registry	10.6
	[51]	1130	Longitudinal	14.7

Multiple system atrophy (MSA)	[15]	26	Cross-sectional	81
	[51]	34	Longitudinal	54.6

Progressive supranuclear palsy (PSP)	[15]	26	Cross-sectional	11
	[51]	30	Longitudinal	13.3

Corticobasal degeneration (CBD)	[15]	14	Cross-sectional	7
	[51]	11	Longitudinal	0

Vascular parkinsonism (VP)	[15]	38	Cross-sectional	26
	[51]	83	Longitudinal	18.3

Dementia with Lewy bodies (DLB)	[15]	32	Cross-sectional	31
	[51]	14	Longitudinal	21.4
	[52]	26	Cohort	42

**Table 2:** Diagnostic approaches to detect orthostatic hypotension in parkinsonian patients.

Diagnostic work-up	Types	Reference
Physical examination	Head-up tilt-table testing (HUT)	—
	Valsalva maneuver	—

Imaging	Cardiac ^123^I-MIBG scintigraphy	—

Heart monitoring	Spectral analysis of the RR interval and systolic blood pressure	[22]

Brain hemodynamics	Transcranial Doppler (TCD)	—
	Diamox injection	[40]
	Carbon dioxide test	[8]
	Cold pressure test (CPT)	[41]

Questionnaire	SCOPA-AUT	[30]
	COMPASS	[31]
	NMSS (Completed by physicians)	[53]
	Freiburg Questionnaire	[54]
	Autonomic dysfunction in PD	[55]
	Orthostatic Grading Scale	[27]
	Hobson Scale	[56]
	Mathias et al. (L-threo-DOPS)	[28, 29]
